# The pro‐inflammatory response of macrophages regulated by acid degradation products of poly(lactide‐co‐glycolide) nanoparticles

**DOI:** 10.1002/elsc.202100040

**Published:** 2021-05-12

**Authors:** Shufang Ma, Xinxing Feng, Fangxiu Liu, Bin Wang, Hua Zhang, Xufeng Niu

**Affiliations:** ^1^ Department of Rheumatology The Fourth Central Hospital of Baoding City Baoding P. R. China; ^2^ Endocrinology and Cardiovascular Disease Centre Fuwai Hospital National Center for Cardiovascular Diseases Chinese Academy of Medical Sciences and Peking Union Medical College Beijing P. R. China; ^3^ Department of Respiratory Medicine The Fourth Central Hospital of Baoding City Baoding P. R. China; ^4^ Department of Pathology Medicine The Fourth Central Hospital of Baoding City Baoding P. R. China; ^5^ Department of Cardiology The Fourth Central Hospital of Baoding City Baoding P. R. China; ^6^ Research Institute of Beihang University in Shenzhen Shenzhen P. R. China

**Keywords:** inflammation, macrophage, PLGA, polarization, THP‐1

## Abstract

Poly(lactide‐co‐glycolide) (PLGA) shows great potentials in biomedical applications, in particular with the field of biodegradable implants and control release technologies. However, there are few systematic and detailed studies on the influence of PLGA degradation behavior on the immunogenicity. In this study, in order to develop a method for dynamically assessing the immunological response of PLGA throughout the implantation process, PLGA particles are fabricated using an o/w single‐emulsion method. The physicochemical characterizations of the prepared PLGA particles during in vitro hydrolytic degradation are investigated. Then, a series of immunological effects triggered by PLGA by‐products formed with degradation process are evaluated, including cell viability, apoptosis, polarization and inflammatory reaction. THP‐1 human cell line is set as in vitro cell model. Our results show that PLGA degradation‐induced acid environment decreases cell viability and increases cell apoptosis, which is a potential factor affecting cell function. In particular, the macrophages exhibit up‐regulations in both M1 subtype related surface markers and pro‐inflammatory cytokines with the degradation process of PLGA, which indicates the degradation products of PLGA can convert macrophages to the pro‐inflammatory (M1) polarization state. All these findings provide the mechanism of PLGA‐induced inflammation and lay the foundation for the design of next‐generation PLGA‐based biomaterials endowed with immunomodulatory functions.

## 1 INTRODUCTION

In recent decades, biodegradable polymers are employed in a variety of medical applications, e.g. carrier for drug delivery system, equipment for orthopedic surgery, stent for tissue engineering, etc. [1, 2, 3]. Biodegradable polymers presently used as tissue engineering scaffolds can be grouped into naturally derived polymers (e.g. hyaluronic acid, collagen, gelatin, alginate, etc.) and synthetic polymers (e.g. poly(ε‐caprolactone), poly(lactic acid) (PLA), poly(glycolic acid) (PGA), etc.) [4, 5]. Compared with natural polymers, biodegradable synthetic polymers have more excellent intrinsic properties such as good mechanical performance, satisfactory batch uniformity, customizable degradation kinetics, and ease of chemical modification, which arouse extensive concern and become one of the most promising field in biomaterial research [6, 7, 8]. However, there are still many problems associated with the application of biodegradable synthetic polymers. Abbott Vascular put a stop to sales of the absorb bioresorbable vascular scaffold in 2017 and the subsequent findings indicate this poly(l‐lactic acid)‐based scaffold increases the risk of thrombosis and restenosis [9, 10]. One of the major concerns is the potential adverse reactions caused by accumulation of by‐products during degradation. Such concerns are exacerbated by the results observed in clinical application of poly(lactide‐co‐glycolide) (PLGA), where serious inflammatory reactions and poor biological activities were reported [11, 12, 13]. Therefore, the evaluation on the biosafety and immunogenicity related with biodegradable synthetic polymers and their degradation products is necessary.

The biocompatibility of biomaterials strongly depends on the response of immune system [14, 15]. It is generally recognized that the host response to implanted biomaterials includes sequential overlapped stages which involves protein adsorption, cell recruitment, acute/chronic inflammation, foreign body response (FBR), granuloma tissue growth and fibrous tissue formation [16]. Various immune cells play their roles in response to the chemical signals produced during this procedure. As the largest number of cells in immune system, neutrophils first infiltrate the tissue/implant interface and dominate in acute inflammation [17]. Monocytes recruited from peripheral circulation migrate to the specific tissue site, where they differentiate into macrophages and dendritic cells, participate in phagocytic clearance and antigen‐presenting, and contribute to the chronic inflammation [18, 19, 20]. Under the influence of interleukin 4 (IL‐4) and interleukin 13 (IL‐13) secreted by mast cells and T helper cells (Th), macrophages fuse into foreign body giant cells in FBR, which recruit and activate tissue repair cells to form fibrous tissue layer around biomaterials eventually [21].

Among the multiple immune cell types, monocytes and macrophages are considered not only as the first line intervening in implantation of biomaterials, but also as key players in homeostasis and all stages of tissue remodeling [22]. Recent research also shows that as the first biological behavior following implantation, inflammatory reaction is closely related to monocyte‐derived macrophage activation [23, 24]. Through interacting with lymphocytes, the activated macrophages are polarized into two major subtypes based on their response to different environmental factors, namely M1 (classically activated macrophages) and M2 (alternatively activated macrophages) [25, 26]. Specifically, Th1 derived signals direct M1 polarization, which in turn enhance Th1 recruitment by secreting IL‐12 and chemokine (C‐X‐C motif) ligand 10. M1 subtype macrophages are responsible for chronic inflammatory diseases, which can secrete pro‐inflammatory cytokines tumor necrosis factor (TNF‐α) and IL‐1β, express typical surface markers C‐C chemokine receptor type 7 (CCR7) and CD86, and prevent foreign body intrusion at the early stage. On the other hand, IL‐4 and IL‐13 released from Th2 induce M2 polarization, which in turn promoting Th2 differentiation by producing chemokine (C‐C motif) ligand (CCL) 17 and CCL 22. M2 subtype macrophages are responsible for immunosuppression, which can produce anti‐inflammatory cytokines IL‐10, express the related surface marker CD206, and encourage tissue regeneration in the later period [27, 28]. Hence, to further promote the application of biodegradable synthetic polymers, it is important to sufficiently discuss the exact regulatory mechanism for such polymers and their degradation products on cellular processes of macrophages.

In this study, we systematically investigate the degradation behavior of PLGA particles, as well as its degradation rate‐dependent in vitro biocompatibility and immunogenicity on human inflammatory cells. Based on the similarity to primary monocytes and macrophages in biology, THP‐1 human monocytic leukemia cell line was used. The present research aims to provide new insights into PLGA‐based biomaterials for further clinical application, so that it can obtain acceptable immunoreaction while maintaining function upon implanted.

## 2 MATERIALS AND METHODS

### 2.1 Materials and cells

PLGA (50:50 Resomer RG 503H, inherent viscosity: 0.32–0.44 dL/g) was purchased from Evonik (Essen, Germany). 1,4‐dioxane was obtained from Sinopharm Group Co. Ltd. (Beijing, China). RPMI 1640 with HEPES, fetal bovine serum (FBS) and penicillin‐streptomycin from Gibco (GrandIsland, USA) was used for in vitro cell culture. Lipopolysaccharide (LPS), tetrahydrofuran (THF) and phorbol ester (PMA) were from Sigma‐Aldrich Co., Ltd. (Shanghai, China). Cell counting kit‐8 (CCK‐8) was purchased from Dojindo Molecular Technologies, Inc., (Shanghai, China). Apoptotic kit was purchased from Roche (Basel, Switzerland). CCR7 antibodies for immunofluorescence (IF) and fluorescence‐activated cell sorting (FACS) were provided by Abcam (Cambridge, UK) and BD (New Jersey, USA), respectively. Secondary antibodies Alexa Fluor 594 and 4′,6‐diamidino‐2‐phenylindole (DAPI) were from ZSGB‐BIO (Beijing, China). TRIZOL solution was got from Invitrogen (Waltham, USA), cDNA Synthesis Kit was obtained from Yeasen (Shanghai, China). Phosphate‐buffered saline (PBS) and bovine serum albumin (BSA) were ordered from Solarbio Science & Technology Co., Ltd. (Beijing, China). Interferon gamma (IFN‐γ), enzyme‐linked immunosorbent assay (ELISA) kits for tumor necrosis factor‐α (TNF‐α) and interleukin‐1β (IL‐1β) were supplied by R&D Systems Inc. (Minneapolis, USA). Human monocytic THP‐1 cell line was obtained from the American Type Culture Collection (Manassas, USA).

### 2.2 PLGA particles preparation

PLGA particles were fabricated using an o/w single‐emulsion method. Briefly, 0.1 g of PLGA was added into 4 mL of dichloromethane and stirred for 1 h to dissolve PLGA thoroughly. Then, this PLGA solution was gradually added into 200 mL of 1% w/v PVA solution under stirring. This solution was stirred at room temperature for 3 h to evaporate dichloromethane, and the solid particles were collected by centrifugation. The resultant particles were washed three times with double‐distilled water and lyophilized for 2 days.

A scanning electron microscope (SEM) (FEI Quanta 250 FEG) was used to observe the surface appearance of particles. The samples were mounted onto metal stubs, coated with a layer of platinum, and then observed microscopically.

### 2.3 PLGA particles degradation behavior

After being sterilized by UV for 6 h, the PLGA particles were washed 3 times with 75% ethanol solution and PBS successively, followed by soaking in 25 mL of serum‐free RPMI 1640 (containing 1% penicillin‐streptomycin) and incubating in cell culture conditions (37°C, 5% CO_2_) to study the degradation behavior. The samples were obtained at the predetermined time intervals (week 0, 1, 2, and 3) for pH, mass loss and molecular weight measurements.

**PRACTICAL APPLICATION**

The potential application of this research lies in the immunomodulation of biodegradable polymers. Our findings provide the mechanism of PLGA‐induced inflammation and lay the foundation for the design of next‐generation PLGA‐based biomaterials endowed with immunomodulatory functions.

As for pH value of degradation medium, the obtained mixture was centrifuged, and the supernatants were harvested under aseptic conditions and stored at 4°C after filtering. The pH value of PLGA particles extract was measured using a pH meter (Leici PHS‐3C, China). Meanwhile, the PLGA particles were recovered from culture medium for subsequent experiments.

As for mass loss analysis, the PLGA samples were detected by weighing the precipitates after washing 3 times with double‐distilled water and freeze‐drying for 48 h. The mass loss rate was then calculated as follows: Mass loss (%) = (*W_i_* – *W*_f_)/*W*_i_ × 100%, where *W*_i_ is the initial mass while *W*_f_ is the final mass of the degraded PLGA particles. Meanwhile, the samples were resolved in tetrahydrofuran (THF) at a concentration of 4 mg/mL. The molecular weight distribution was determined by gel permeation chromatography (GPC, Agilent, USA) with THF as mobile phase, 1 mL/min flow rate, 37°C column temperature and 20 μL injection volume for each analysis.

### 2.4 Cell culture and differentiation

The THP‐1 cells were used as in vitro model. The cells were maintained at a concentration of 2∼5 × 10^5^ cells/mL in RPMI 1640 medium (containing 1% penicillin‐streptomycin) supplemented with 10% FBS at 37°C under a humidified condition with 5% CO_2_. The culture media was replaced every 2 days with fresh media and only early passage cells (p3‐5) were used throughout the whole experiment. For PMA‐induced differentiation, the THP‐1 cells were treated with 50 ng/mL PMA for 48 h to differentiate into M0 macrophages for subsequent study.

### 2.5 Macrophage polarization induction

In the present study, M0 macrophages as above were treated with 50 ng/mL LPS plus 10 ng/mL IFN‐γ to develop into M1 macrophages. Negative control group was set without LPS plus IFN‐γ induction, while experiment groups Ctr (pH 7.28), pH 7.0, pH 6.7 and pH 6.4 were co‐cultured with PLGA particles extract media in various pH values. Detections for macrophage viability, apoptosis, morphology, surface maker expression, cytokine secretion and relative mRNA expression were carried out after another 2 and 4 days of co‐culture (5% CO_2_ at 37 °C), respectively.

### 2.6 Cell viability

CCK8 assay was used to evaluated the impact of PLGA degradation on cell viability, THP‐1‐derived macrophages were cultured with RPMI 1640 medium (pH 7.28) or PLGA extracts at different pH values (7.0, 6.7 and 6.4) for 2 and 4 days. Then, 100 μL samples from each group were transferred into each well of a new 96‐well plate and supplemented with 10 μL CCK8 reagent. After an additional 3 h of incubation at 37°C, 5% CO_2_, the optical density was detected at 450 nm in a microplate reader (ThermoFisher, USA). The viability of cells was calculated as follows: Cells relative growth (%) = *OD*_t_/*OD*_c_ × 100%, where *OD*_t_ and *OD*_c_ indicated the optical density of test and control groups, respectively. For cellular morphology, the corresponding macrophages were observed via light microscope (Olympus, Japan).

### 2.7 Cell apoptosis

The apoptotic kit was used to detect the apoptotic induction effect of pH on THP‐1‐derived macrophages. After 2 and 4 days of exposure to the indicated culture media or PLGA extracts mentioned above, the macrophages were harvested and washed using PBS. The cells were then stained with Annexin‐V APC (1:100) for 25 min in ice, followed by adding 5 μL 7‐AAD to each tube and incubating for another 5 min at room temperature in the dark. After being washed with PBS, the cells were resuspended for the apoptosis rate analysis via the flow cytometer (BD FACSCanto Plus, USA).

### 2.8 Immunofluorescence staining assays

For qualitative analysis of CCR7 (M1 macrophage surface marker) expression level, immunofluorescence staining was performed following the protocol. The cells were treated with predetermined PLGA extracts at 1 × 10^5^ cells/mL in a glass bottom dishes. After being harvested at appropriate time points, the macrophages were fixed, permeabilized and blocked, then incubated with the primary antibodies CCR7 at 1:100 in 1%BSA/PBS for 1 h at normal temperature. Afterwards, the secondary antibodies Alexa Fluor 594 (1:200) were added, followed by incubating with the macrophages away from direct light at 4°C overnight. After PBS wash and cell nucleus staining with DAPI, the cellular fluorescence distribution was observed and imaged through the confocal laser scanning microscope (Leica SP8, Germany).

### 2.9 Flow cytometry analysis

For the determination of macrophages polarization state, flow cytometry was used to quantitatively analyze the expression levels of macrophage surface marker, namely CCR7 and CD206 (M2 macrophage surface marker). In brief, the PLGA extracts pretreated macrophages were obtained at the end of 2 and 4 days culture, followed by digesting, blowing and detaching from the culture plates. Then, the collected macrophages were blocked with 1% BSA/PBS for 40 min, incubated in PBS containing antibodies of CCR7 and CD206 for additional 40 min at normal temperature in the dark. After washing steps, the cells were resuspended in 1% BSA and examined via the flow cytometer.

### 2.10 ELISA

To examine the effect of PLGA degradation products on inflammatory response of THP‐1‐derived macrophages, the sandwich enzyme‐linked immunosorbent assay (ELISA) principle was performed to detect the production of pro‐inflammatory cytokines, namely TNF‐α and IL‐1β. After being co‐cultured with PLGA extracts under different pH values for the indicated durations as mentioned above, the corresponding culture medium were obtained and frozen immediately at –20°C before the assay was conducted. Enzyme‐linked immunosorbent was carried out as described in the manufacturer's protocols of the ELISA kits. The concentrations of TNF‐α and IL‐1β were determined based on the absorbance of test samples and the standard curves constructed at the same conditions.

### 2.11 Real‐time PCR

To confirm the inflammatory response of macrophages in gene level, the real‐time quantitative polymerase chain reaction (RT‐qPCR) analysis was performed to double‐check the relative mRNA expressions, TNF‐α and IL‐1β. After predetermined durations of incubation with PLGA extracts using the same method described, total RNA was isolated and extracted from the collected macrophages by TRIZOL solution, and then quantified via the micro volume spectrophotometer (Jenway, UK). The cDNA was reverse transcribed and further amplified according to the manufacturer's instructions of cDNA Synthesis Kit (Yeasen, China) using 2 μL total RNA from each group. Afterwards, the real‐time PCR was carried out employing Eco 48 real‐time PCR detection system (PCRmax, UK). The quantified data of target mRNA TNF‐α and IL‐1β were done using ΔΔCt method and normalized to housekeeping gene GAPDH. The primers applied in this study are given in Table 1.

**TABLE 1:** Primers applied for RT‐qPCR

Target gene	Direction	Sequence (5′‐3′)
GAPDH	Forward	GGAGCGAGATCCCTCCAAAAT
	Reverse	GGCTGTTGTCATACTTCTCATGG
TNF‐α	Forward	CGAGTCTGGGCAGGTCTA
	Reverse	GTGGTGGTCTTGTTGCTTAA
IL‐1β	Forward	CCCTCTGTCATTCGCTCCC
	Reverse	CACTGCTACTTCTTGCCCCC

### 2.12 Statistics

Individual experiments involving statistical analysis in this study were consisted of three or more parallel samples. All the data were expressed as means ± standard deviation (SD) and performed by Prism 7 (GraphPad Software, USA). The differences among means were considered the level of significance at *P < 0.05, **P < 0.01.

## 3 RESULTS AND DISCUSSION

### 3.1 Characterization of PLGA particles

**FIGURE 1:**
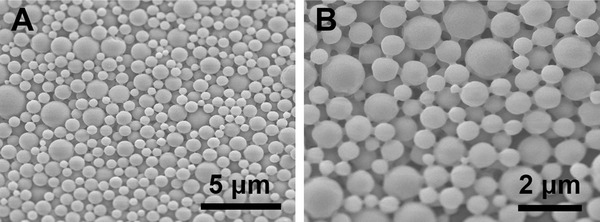
Surface morphology of PLGA nanoparticles: (A) 5000×; (B) 10,000×

To fabricate PLGA particles with small size, a double‐emulsion solvent extraction/evaporation method was used in the present research. Figure 1 is the surface appearance of the prepared particles. The particles were spherical in shape and had a regular surface without any cracks. The particle size distribution was in the range of tens to hundreds of nanometers based on SEM observation, which was favorable for the following hydrolytic degradation.

### 3.2 In vitro hydrolytic degradation of PLGA particles

**FIGURE 2:**
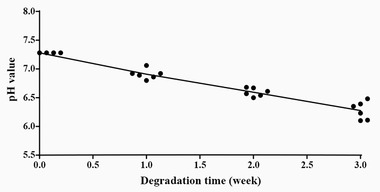
pH value changes of the extracts during in vitro degradation. PLGA was set in RPMI 1640 media for 3 weeks and the pH value of media was tested each week. (n = 6)

To analyze whether the microenvironment of further cell culture media altered, the pH values were tested. As shown in Figure 2, when most of PLGA degradation products were being dissolved, the pH value dropped from 7.28 (RPMI 1640 media) to an average of 6.4 up to week 3, due to the accumulation of acidic degradation products lactic acid and glycolic acid. Based on these results, we hypothesis that media around PLGA in body may be acidic and later the media we used for culturing with macrophage was also acidic to certain extent.

Table 2 summarizes the degradation behavior of PLGA particles. The mass loss rate showed a quick increase to an average of 5.92% in the first week, and remained relatively a steady and slower growth phase to 8.37% afterward by 2 weeks exposure period, continued by a burst rise to 18.93% up to week 3, which was corresponding to pH changes in Figure 2. Besides, the number‐average molecular weight (M_n_) and weight‐average molecular weight (M_w_) associated with mass loss appeared a gradual decrease to an average of 1.36 and 3.84 kDa, respectively, by the end of degradation‐testing program, which followed similar trends with mass loss rate.

**TABLE 2:** The degradation behavior of PLGA particles

Degradation time	Mass loss rate (%)	M_n_ (kDa)	M_w_ (kDa)	M_w_/M_n_ (PD)	Code
Week 0	0	5.46 ± 1.33	13.30 ± 1.44	2.51 ± 0.49	Ctr
Week 1	5.92 ± 1.57	3.59 ± 1.07	10.76 ± 4.02	2.97 ± 0.23	pH 7.0
Week 2	8.37 ± 2.90	3.16 ± 0.41	7.17 ± 0.51	2.29 ± 0.27	pH 6.7
Week 3	18.93 ± 3.44	1.36 ± 0.30	3.84 ± 1.20	3.04 ± 1.61	pH 6.4

The main degradation form of PLGA is hydrolysis, which carried out through bulk erosion mechanism [29]. The proportion of PLA and PGA units in the copolymer played an important role in the degradation. Since PGA units degrades faster than PLA units due to the stronger hydrophilicity of GA‐GA bonds than those of LA‐LA and GA‐LA bonds, the mass and molecular weight decreased rapidly in the initial degradation. In the following week 2, water molecules further infiltrate PLGA, and the degradation occurred mainly in PLA. The mass loss and molecular weight declined relatively slowly. In week 3, the ester bond in PLA units were hydrolyzed, and oligomers or monomers were formed, which leaded to a rapid drop in molecular weight.

Besides, the increasing acidic degradation products released from PLGA lowered the local pH, which also contributed to the relatively increased degradation rate during this period and resulted in the autocatalytic hydrolysis effects. Thinking that the degradation products of PLGA in vivo were gradually transformed and metabolized by local cells, it is particularly important to study the effect of degrading medium with different pH values on immune cell reaction. In the following study, we chose PLGA extracts including representative pH values (7.0, 6.7, and 6.4) at the end of each observed week, to study the PLGA‐induced inflammatory response on THP‐1‐derived macrophages.

### 3.3 The effect of PLGA extracts on macrophages viability

**FIGURE 3:**
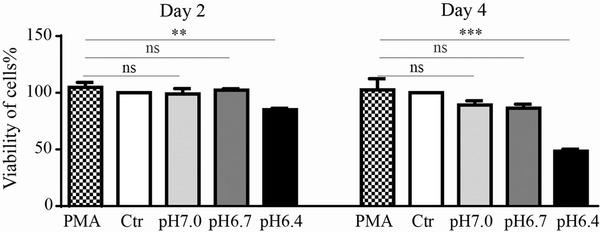
The viability of THP‐1‐derived macrophages. CCK‐8 test of THP‐1‐derived macrophages were assayed on day 2 and 4 in response to PLGA extracts with different pH values or RPMI 1640. All THP‐1 cells were treated by PMA (50 nM) for 24 h and developed into macrophages, after that cells were cultured with media (PMA), pH 7.28 extracts (Ctr), pH 7.0 extract (pH 7.0), pH 6.7 extract (pH 6.7) and pH 6.4 extract (pH 6.4) for 2 or 4 days. Data are presented as mean ± SEM. **P < 0.01; ***P < 0.001; ns, not significant. Each experiment was independently repeated at least three times

In order to examine whether pH alteration in media modulated by PLGA extracts play a role on the function of THP‐1‐derived macrophages, we evaluated cell viability after 2 or 4 days of incubation in PLGA extract media. THP‐1‐derived macrophages were induced by PMA by 24 h (M0 subtype). As illustrated in Figure 3, the viability of macrophages was almost unaffected by the degradation of PLGA for 2 days’ treatment. However, when the cell culture time increased to 4 days, we observed a significant reduction in the pH 6.4 group—its viability was less than 50%, while the other groups remained similar high viability. This result hit a clue that if it reacted enough time, PLGA degradation may influenced the viability of macrophages due to the decreasing pH value, especially when the medium pH was less than 6.5.

### 3.4 The effect of PLGA extracts on macrophage apoptosis

**FIGURE 4:**
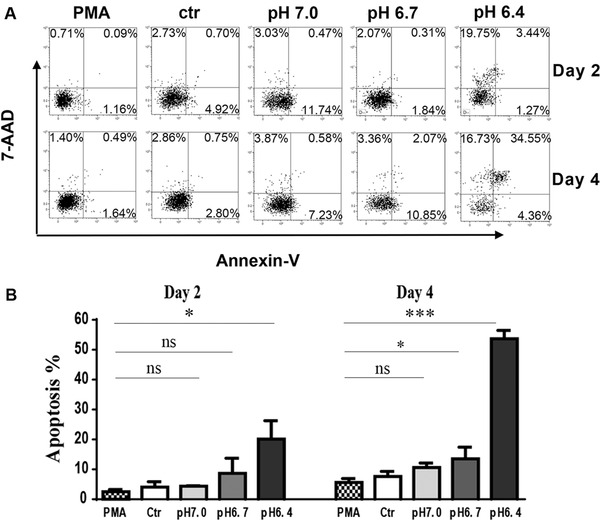
The apoptosis of THP‐1‐dereived macrophages. All THP‐1 cells were treated by PMA (50 nM) for 24 h and developed into macrophages, after that cells were cultured with media (PMA), pH 7.28 extracts (Ctr), pH 7.0 extract (pH 7.0), pH 6.7 extract (pH 6.7) and pH 6.4 extract (pH 6.4) for 2 or 4 days. (A) Annexin V plus 7‐PI staining by flow cytometry on day 2 and 4, respectively, a representative data from one experiment. (B) statistical results on apoptotic THP‐1‐derieved macrophages as Annexin V+ cells. Data are presented as mean ± SEM. *P < 0.05; ***P < 0.001; ns, not significant. Each experiment was independently repeated at least three times

Since the decreased cell viability was often associated with the damage of cytomembrane integrity, we conducted the cell apoptosis experiment at the same time harvested in viability test. As shown in Figure 4, there were no big differences between PMA, Ctr, pH 7.0, pH 6.7 groups, their apoptosis rates were all less than 5%. However, we observed that there was a strikingly elevated Annexin V+ cells in pH 6.4 groups both in day 2 and 4. Seriously, the apoptotic ratio was touched approximately 50% in day 4, far greater than other groups. In sum, these results demonstrated that the influence of PLGA extracts on cell viability was attributed to the low pH following the degradation, which weakened the cytomembrane integrity and caused cell apoptosis. This may suggest that acidic media modulated by PLGA extracts were harmful to macrophage growth. Since pH 6.4 medium was too toxic for cell survival and could inevitably interfere with our study, we excluded this group in later experiments.

### 3.5 The effect of PLGA extracts on macrophage morphology

**FIGURE 5:**
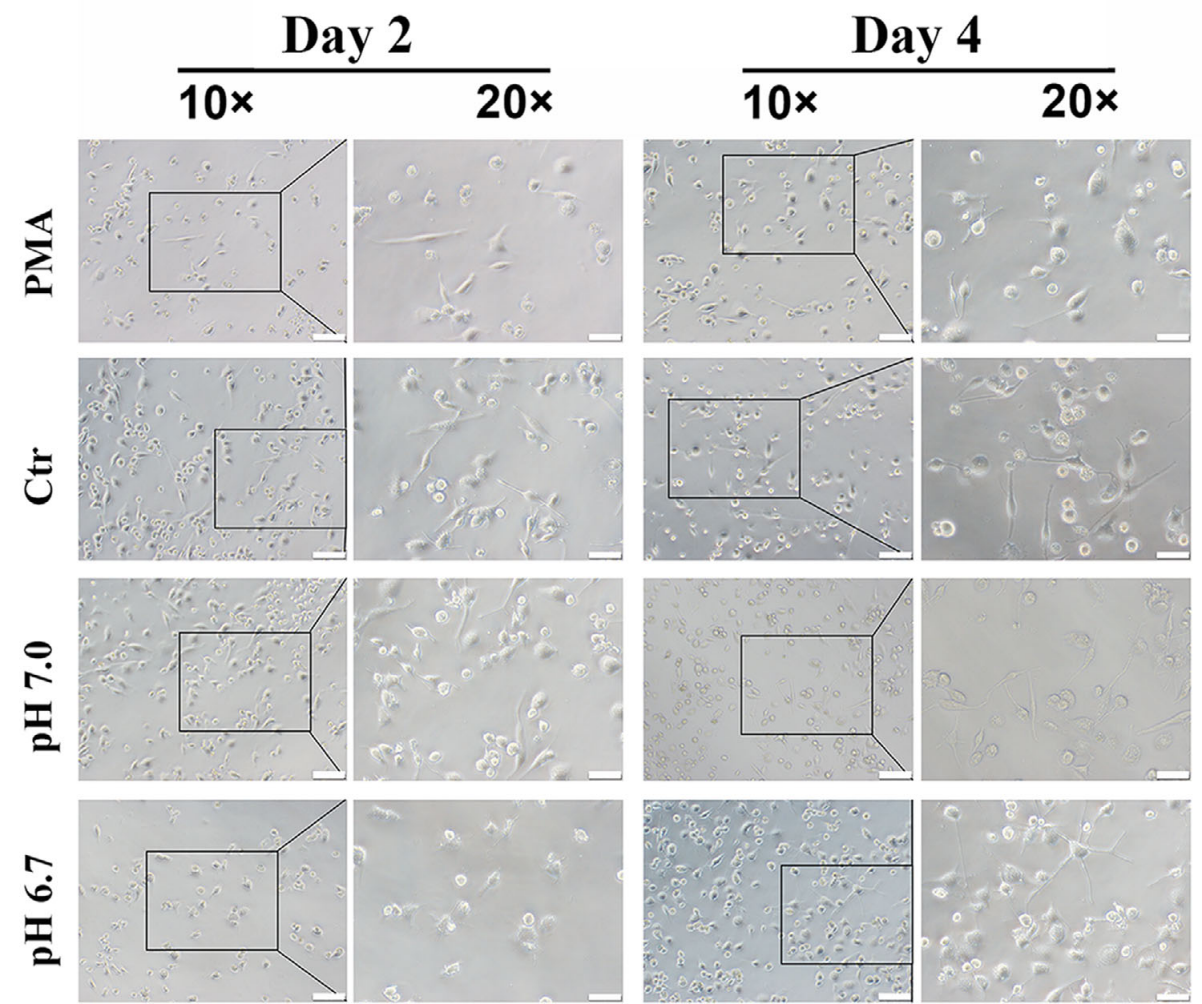
Cytomorphology changes of THP‐1‐derived macrophages. All THP‐1 cells were treated by PMA (50 nM) for 24 h and developed into macrophages, after that cells were cultured with media (PMA), pH 7.28 extracts (Ctr), pH 7.0 extract (pH 7.0), and pH 6.7 extract (pH 6.7) for 2 or 4 days. Scale bar: 100 μm in 10× and 50 μm in 20 ×

The morphological differences of macrophages with a decreased pH during culture time were observed. After stimulated by PMA, the differentiated M0 macrophages were in an adherent state with irregular shape. No significant difference was found on the macrophage morphology between high pH group (pH 7.0) and control group (Ctr) after 2 days of culture. However, the density of adhesive macrophages went down with pH value especially in pH 6.7 group (with few macrophages attached). Furthermore, few macrophages in PMA group were shown both oblate and fusiform shape at the same time, which implied a mixture of M1 and M2 macrophages after differentiation, whereas the macrophages in PLGA extracts‐treated groups were shown less elongated pseudopodia and converted to a more flat and round state as decreasing pH or prolonging culture duration (Figure 5).

The cell‐microenvironment interactions impact cell morphology. The pancake appearance is a typical morphology of M1 macrophages [21]. Hence, changes in the shape of THP‐1‐derived macrophages are supposed to be attributed to the M1 polarization state transformation.

### 3.6 The effect of PLGA extracts on macrophage polarization

**FIGURE 6:**
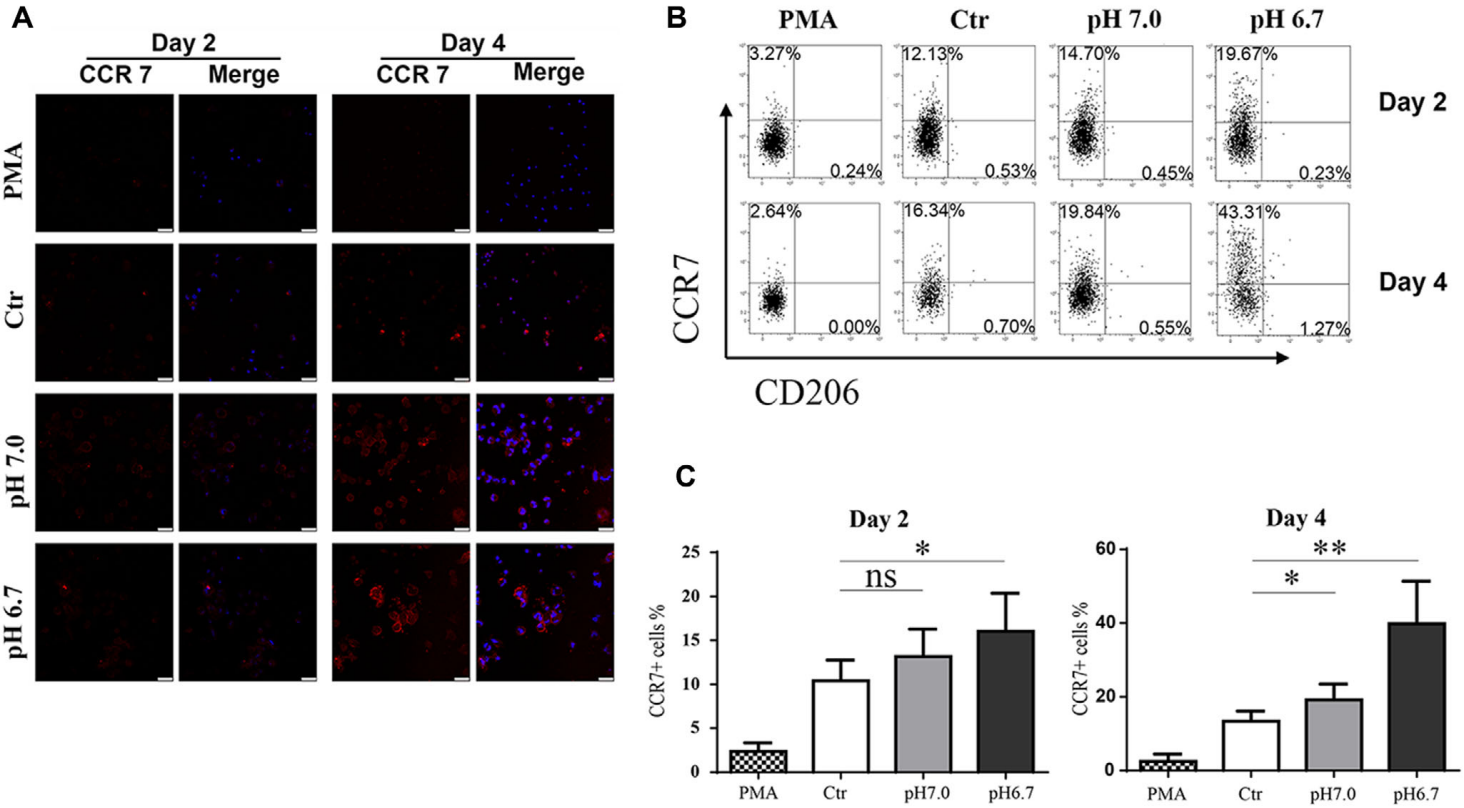
Quantitative identification on the polarization of THP‐1‐derived macrophages. All THP‐1 cells were treated by PMA (50 nM) for 24 h and developed into macrophages, after that cells were cultured with media (PMA), pH 7.28 extracts (Ctr), pH 7.0 extract (pH 7.0), and pH 6.7 extract (pH 6.7) for 2 or 4 days. (A) Immunofluorescent staining of THP‐1‐derived macrophages. CCR7 (red fluorophore) indicated M1 macrophages, nuclei were stained with DAPI (blue fluorophore). Scale bar: 75 μm. (B) Surface markers CCR7 and CD206 expression on THP‐1‐derived macrophages determined by flow cytometry, a representative data from one experiment. (C) The statistical analysis of CCR7 expression calculated from the flow cytometric plots. Data are presented as mean ± SEM. *P < 0.05; **P < 0.01; ns, not significant. Each experiment was independently repeated at least three times

The influence of pH on macrophage phenotype shift was also investigated by immunofluorescence staining assay. CCR7 is a surface marker of pro‐inflammatory macrophage subtype (M1). As shown in Figure 6A, macrophages cultured in the presence of PLGA extracts showed obviously more extensive staining area of CCR7 compared with Ctr groups. Moreover, CCR7 positively stained cells increased as the pH value decreased. Besides, very few CCR7 expression appeared in non‐LPS/IFN‐γ treated cells from PMA group, which indicated the M0 macrophage state.

To furthermore confirm the effect of PLGA extracts on polarization, we double‐checked the cell surface marker expressions by flow cytometry (Figure 6B and C). Following polarization to M1 subtype (Ctr) as indicated by a significant increased CCR7, the percentages of CCR7 positive macrophages were notably affected by the PLGA extracts with different pH value (pH 7.0 and 6.7). In contrast, M2 macrophage marker CD206 as a control had no significant changes among all groups. The flow cytometer evaluation indicated that CCR7 positive macrophages increased apparently together with the reduction in pH value. The similar results were also found in CD86 test (data not shown). On day 4, the pH 6.7 group had significantly higher in percentage of CCR7^+^ M1‐like macrophages, almost triple than Ctr group, and even 20 times than the untreated M0 macrophages (PMA).

### 3.7 The effect of PLGA extracts on pro‐inflammatory cytokine secretion

**FIGURE 7:**
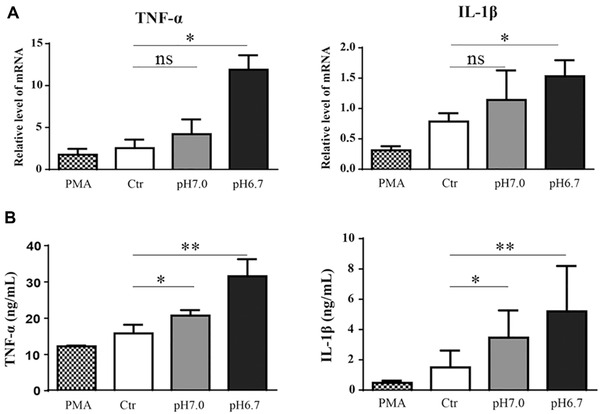
PLGA degradation products regulated inflammatory responses of THP‐1‐derived macrophages. All THP‐1 cells were treated by PMA (50 nM) for 24 h and developed into macrophages, after that cells were cultured with media (PMA), pH 7.28 extracts (Ctr), pH 7.0 extract (pH 7.0), and pH 6.7 extract (pH 6.7) for 4 days. (A) mRNA expression by PCR and (B) the secreted protein level of TNF‐α and IL‐1β by ELISA in THP‐1‐derived macrophages. Data are presented as mean ± SEM. *P < 0.05; **P < 0.01; ns, not significant. Each experiment was independently repeated at least three times

To further explore the immunomodulation of PLGA extracts on THP‐1‐derived macrophages through pH value alteration, we inspected cytokines secretion and relative mRNA expression. TNF‐α and IL‐1β, the most prominent pro‐inflammatory cytokines were used as the M1 macrophage markers [30]. Figure 7A shows that the mRNA levels of both TNF‐α and IL‐1β were nicely up‐regulated in response to PLGA extracts with pH 7.0 and pH 6.7 in day 4. The observations above were further confirmed in secreted cytokines tested by ELISA (Figure 7B). Both TNF‐α and IL‐1β secreted by macrophages were enhanced after co‐culturing with different PLGA extract medium. There was an apparent pH value dependent from Ctr to pH 6.7 group, the more acidic the more cytokine production, representing an elevated inflammatory effect. Based on the similar pattern between RT‐qPCR and ELISA, it indicated that PLGA by‐products could induce the inflammatory response of THP‐1‐derived macrophages through up‐regulating the gene expression of pro‐inflammatory cytokines.

In brief, the alterations in cytokine and genetic levels, further confirmed the role played by PLGA in macrophages polarization states during its degradation, which implied a gradual transition into the more M1 subtype polarization state. It suggested that the inflammatory response caused by PLGA degradation was enhanced in a by‐product dose‐dependent manner to some extent.

In summary, the M1 subtype‐specific surface marker CCR7 showed the presence of inflammatory cells and the enhancement of their presence under the PLGA extracts condition with decreased pH values. The cellular morphology was also affected by the degradation by‐products of PLGA particles, and changed to a more pancake‐like shape. Since overexpressed in almost every inflammatory disease, TNF‐α and IL‐1β are used in our study. After PLGA extracts (pH 7.0) treatment, the LPS/IFN‐γ‐stimulated M1 subtype THP‐1 cells expressed a larger amount of TNF‐α and IL‐1β found in both ELISA and RT‐qPCR assays, and we even observed the greater enhancement in pH 6.7 group, which could be explained by the effect of acid micro‐environment. It indicated that the PLGA by‐products treatment promoted the local macrophage population into M1 subtype as the degradation process, which triggered a more ampliative inflammatory reaction. This may be attributed to the PLGA properties that are able to undergo rapid degradation to glycolic acid and lactic acid, leading to the formation of an acidic extracellular microenvironment and the drop in intracellular pH through Na^+^‐HCO_3_^−^ co‐transporter and Cl^−^/HCO_3_^−^ exchanger [31]. Afterward, the phosphorylation of p38 mitogen‐activated protein kinases (MAPKs) and extracellular signal‐related kinase 1/2 (ERK1/2) caused the activation of NF‐κB in THP‐1‐derived macrophages, followed by the up‐regulation of inducible nitric oxide synthase (iNOS) and cyclooxygenase‐2 (COX‐2), and eventually leading to the increasing production of pro‐inflammatory cytokines, including TNF‐α and IL‐1β. The abnormalities in the production or function of TNF‐α and IL‐1β in turn increased the severity of inflammatory response [32]. Hence, this underlying mechanism has a greater potential to trigger macrophage activation and increase the percentage of M1 macrophages. A more precise molecular mechanism of PLGA on macrophage polarization and inflammation is needed in further study.

## 4 CONCLUDING REMARKS

Biodegradable PLGA particles are successfully fabricated by double‐emulsion solvent extraction/evaporation method. Co‐culture of THP‐1 derived macrophages with various pH PLGA extracts during in vitro hydrolytic degradation indicates that PLGA extracts‐treated macrophages presents an up‐regulated M1 macrophage response, and this clear trend is demonstrated in a time and pH‐dependent manner.

## CONFLICT OF INTEREST

The authors have declared no conflict of interest.

## Data Availability

The data that support the findings of this study are available from the corresponding author upon reasonable request.
